# Clinical characteristics and long-term neurodevelopmental outcomes of leukomalacia in preterm infants and term infants: a cohort study

**DOI:** 10.1186/s11689-023-09489-7

**Published:** 2023-08-07

**Authors:** Juan Song, Yuyang Yue, Huiqing Sun, Ping Cheng, Falin Xu, Bingbing Li, Kenan Li, Changlian Zhu

**Affiliations:** 1 Henan Key Laboratory of Child Brain Injury and Henan Pediatric Clinical Research Center, Institute of Neuroscience and Third Affiliated Hospital of Zhengzhou University, Zhengzhou, 450052 China; 2 Department of Neonatology, Children’s Hospital of Zhengzhou University, Zhengzhou, 450018 China; 3 Department of Neonatology, First Hospital of Zhengzhou, Zhengzhou, 450000 China; 4 Center for Brain Repair and Rehabilitation, Institute of Neuroscience and Physiology, Sahlgrenska Academy, University of Gothenburg, 40530 Gothenburg, Sweden; 5 Department of Women’s and Children’s Health, Karolinska Institutet, 17176 Stockholm, Sweden

**Keywords:** Leukomalacia, Newborn, Head MRI, Neurodevelopmental outcomes, Cerebral palsy

## Abstract

**Background:**

Leukomalacia is a serious form of neonatal brain injury that often leads to neurodevelopmental impairment, and studies on neonatal leukomalacia and its long-term outcomes are lacking. The aim of this study was to analyze the clinical manifestations, imaging features, and long-term neurodevelopmental outcomes in preterm infants and term infants with leukomalacia.

**Methods:**

Newborns diagnosed with leukomalacia by head magnetic resonance imaging (MRI) and who were admitted to intensive care units from January 2015 to June 2020 were enrolled. All infants were followed up to June 2022 (2–7 years old), and their neurodevelopmental outcomes were evaluated. The clinical data and long- term outcomes of preterm infants and term infants was analyzed by Chi-square tests.

**Results:**

A total of 218 surviving infants with leukomalacia including 114 preterm infants and 104 term infants completed the follow-up. The major typesof leukomalacia on MRI were periventricular leukomalacia in the preterm group and subcortical cystic leukomalacia in the term group, respectively (χ^2^ = 55.166; *p* < 0.001). When followed up to 2–7 years old, the incidence of neurodevelopmental impairment in the preterm group and term group was not significantly different (χ^2^ = 0.917; *p* = 0.338). However, the incidence of cerebral palsy (CP) in the preterm group was significantly higher (χ^2^ = 4.896; *p* = 0.027), while the incidence of intellectual disability (ID) (χ^2^ = 9.445; *p* = 0.002), epilepsy (EP) (χ^2^ = 23.049; *p* < 0.001), and CP combined with ID andEP (χ^2^ = 4.122; *p* = 0.042) was significantly lower than that in the term group.

**Conclusions:**

Periventricular leukomalacia mainly occurred in preterm infants while subcortical cystic leukomalacia was commonly seen in term infants. Although the long-term neurodevelopmental outcomes of leukomalacia were both poor, preterm infants were more prone to CP, while term infants were more prone to ID, EP, and the combination of CP with ID and EP.

## Introduction

In recent years, the popularization of neonatal intensive care units (NICUs) and rapid progress in intensive care technology have reduced the mortality of premature infants, low-birth-weight infants, and severely asphyxiated infants, but the incidence of neonatal brain injury remains high [1]. Leukomalacia is a severe form of neonatal brain injury that often leads to neurodevelopmental impairment (NDI) such as cerebral palsy (CP), epilepsy (EP), intellectual disability (ID), and aural and visual impairment [2, 3] and thus brings a heavy burden to families and society. Leukomalacia arises from a multifaceted pathogenesis involving hypoxia–ischemia and infection as the primary underlying factors. The progression of leukomalacia involves microglial activation, hypomyelination, astrogliosis, neuronal death, and various other pathways [4]. In developed countries, about 2–8 of every 1000 live births experience leukomalacia [5]. One recent report showed that the incidence of leukomalacia in extremely premature infants in tertiary medical centers in China was 16.7% [6]. However, there is no specific treatment for leukomalacia. Therefore, studies exploring the clinical characteristics and preventive strategies of neonatal leukomalacia are of great importance.

The main pathological types of leukomalacia are periventricular leukomalacia (PVL) and subcortical cystic leukomalacia (SCL) [7]. PVL is characterized by one or more cysts located in the area around the ventricle, and in severe cases even the semi-oval center or the deep subcortical white matter can be involved [8]. The cysts associated with SCL are mainly located in the deep white matter area near the cortex [7, 9]. Although PVL is a common type of leukomalacia in preterm infants, it can also be found in term infants. A recent study in Canada reported that 7.06% of term children with CP are complicated with PVL [10], and in China the morbidity of PVL in term children with CP was reported to be 5.7% [1]. In fact, both types of leukomalacia can occur in both premature and term infants [11].

Up to now, studies on leukomalacia have mainly focused on the clinical features, pathogenesis, prevention, and treatments in premature infants, and only a few studies have looked at leukomalacia in term infants and the differences between preterm infants and term infants. In addition, studies on neurological outcomes in children with leukomalacia are often limited to small sample sizes and short-term follow-up times and do not always involve all types of leukomalacia [1, 12]. Therefore, we carried out a prospective cohort study to analyze the differences in clinical manifestations, imaging features, and long-term neurodevelopmental outcomes between premature infants and term infants with leukomalacia in order to get a better understanding of neonatal leukomalacia and its long-term neurological outcomes.

## Methods

### Study design

This was a double-center prospective cohort study. Newborns diagnosed with leukomalacia by head magnetic resonance imaging (MRI) who were admitted to the Neonatal Department of the Third Affiliated Hospital of Zhengzhou University and Henan Children’s Hospital from January 2015 to June 2020 were enrolled. Infants with inherited metabolic disorders and congenital brain malformations were excluded. All eligible infants were divided into the preterm group (gestational age < 37 weeks) and term group (gestational age ≥ 37 weeks) and followed up to June 2022 (2–7 years old). Clinical characteristics and long-term neurological outcomes of infants with leukomalacia who survived and completed the follow-up were analyzed. This study was approved by the Ethics Committee of the Third Affiliated Hospital of Zhengzhou University, and informed consent was signed by all parents.

### Clinical data

Clinical information including gender, birth weight, gestational age, small for gestational age, 5-min Apgar score, delivery mode, and mechanical ventilation as well as neonatal complications such as apnea (requiring drug intervention or upgrading of oxygen therapy), neonatal seizures, symptomatic hypoglycemia, sepsis, hemodynamically significant patent ductus arteriosus (hs-PDA), intraventricular hemorrhage (IVH), cerebral parenchymal hemorrhage, and purulent meningitis were collected. Symptomatic hypoglycemia was defined as blood sugar below 2.6 mmol/L (47 mg/dl) accompanied by drowsiness, feeding difficulty, and/or seizures [13], and IVH was diagnosed by head ultrasound and classified into four grades according to Papile [14].

### Head MRI

**Fig. 1:**
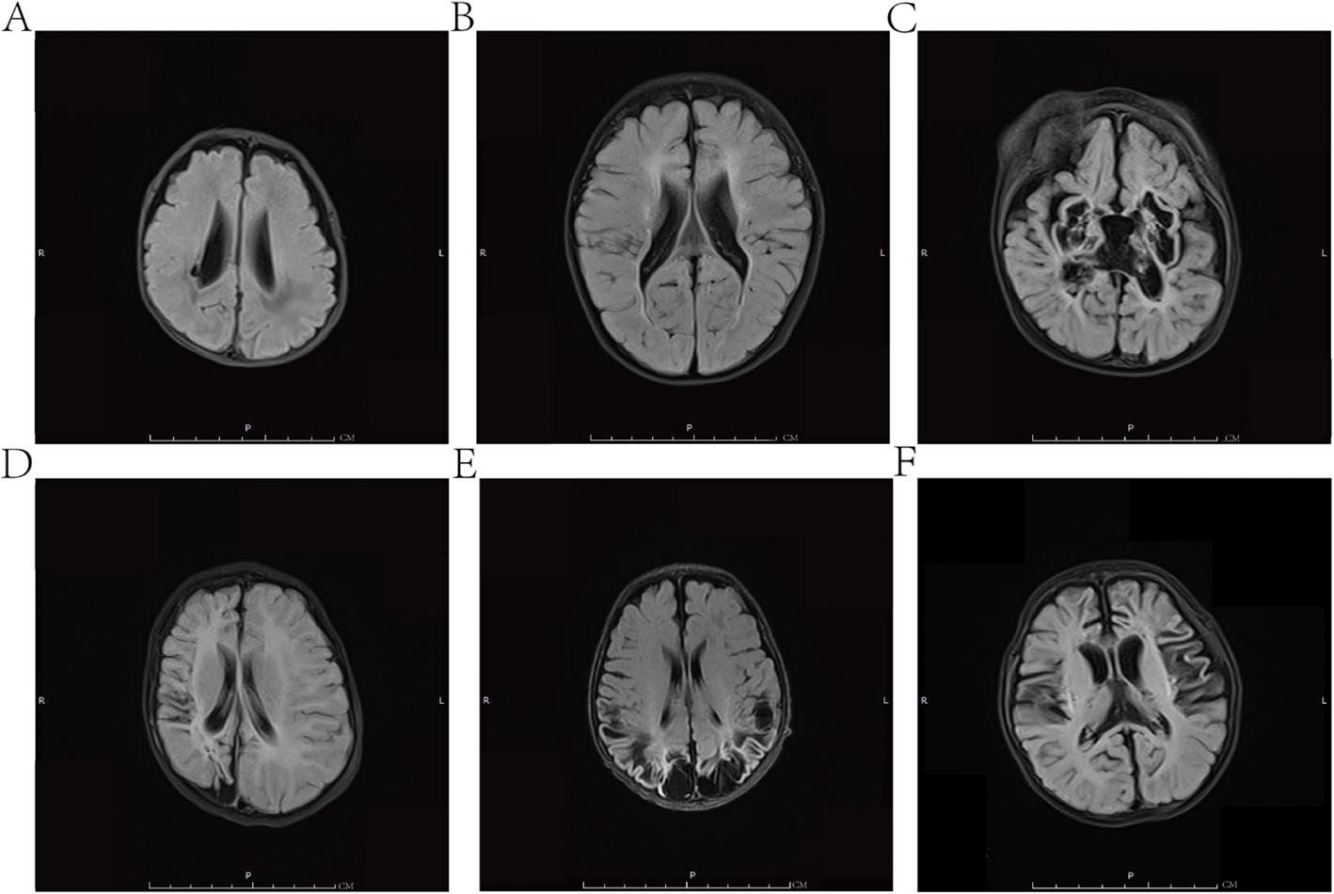
Different types of leukomalacia on head MRI (T2 FLAIR). **A**. Grade I PVL: local changes in the small capsule cavity around the body of the right lateral ventricle. **B**. Grade II PVL: extensive cystic changes around the ventricle, with an irregular ventricular wall and ventricular dilatation. **C**. Grade III PVL: cystic changes in the periventricular and subcortical white matter. **D.** SCL: cysts in the right parietal lobe. **E**. SCL: cysts in the bilateral parietal and occipital lobes. **F**. SCL: cysts in the bilateral frontal and parietal lobes

All infants were routinely screened with cranial ultrasound within 3 days after birth, then on day 7 and weekly thereafter until discharge. Head MRI (GE SIGNA Creator 1.5 T) was performed immediately when cystic lesions were detected on ultrasound, and at 40 weeks of corrected gestational age, and then at 3–6 months intervals. All participants were sedated with phenobarbital sodium and then scanned under pulse oxygen saturation monitoring (breathing support if necessary). A conventional MRI sequence protocol was applied in all of the participants: AX: T_1_WI (TR 3010 ms, TE 14 ms), T_2_WI (TR 4500 ms, TE 99 ms), T_2_ Flair (TR 8000 ms, TE 105 ms). Sag: T_1_WI (TR200 ms, TE2.60 ms). The type, severity, and cyst sites of leukomalacia were evaluated and divided into PVL and SCL by head MRI. In this study, we took into account the classifications of Vries et al. and Choi et al. [15, 16] and classified PVL into three grades. Grade I included local changes in the small capsule cavity around the ventricle (high signal on T2-weighted image and low signal on T2 FLAIR (fluid-attended inversion recovery). Grade II included extensive cystic changes around the ventricle, which could be fused into pieces (irregular ventricular wall or ventricular dilatation). Grade III included cystic changes in the periventricular and subcortical white matter. SCL was defined as cystic changes in the deep subcortical white matter including the frontal lobe, temporal lobe, parietal lobe, occipital lobe, etc., as shown in Fig. 1.

### Follow-up

All eligible infants were followed up for growth and neurological development at the age of 3 months, 6 months, 12 months, 18 months, and 24 months and at least once a year thereafter until June 2022 when they were 2–7 years old. NDI was defined in this study as survival with one or more adverse neurodevelopmental outcomes including CP, ID, EP, vision problems, and deafness. According to the criteria of Bax et al. [17], CP was classified into spastic CP (spastic quadriplegia, diplegia, and hemiplegia) and other CP (dyskinesia, ataxia, hypotonia, and mixed CP). The motor function in CP patients was assessed by the gross motor function classification system (GMFCS) and was classified into independent walking with or without assistance (I–III) and inability to walk independently (IV and V) [18]. Intelligence was evaluated by Griffiths mental development scales-Chinese [19], and ID was defined as a General Quotient score < 70 [20]. EP was diagnosed according to the criteria of the International League Against Epilepsy [21] and was classified as West syndrome, partial seizures, generalized seizures, and mixed seizures. Early vision screening mainly consisted of fundus stereo photography and dynamic visual acuity test by experts. Vision problems were defined as blindness, strabismus, and amblyopia. Early hearing screening consisted primarily of otoacoustic emission and auditory brainstem response, with specialist examination when necessary. Deafness was defined as total or partial hearing loss and the need for hearing aids.

Follow-up was carried out by specialists including neonatologists, neurologists, rehabilitation physicians, ophthalmologists, and otolaryngologists. Follow-up data were obtained through outpatient follow-up records, in-patient electronic medical records (children who were hospitalized for intervention treatment), and remote video and telephone interviews.

### Statistical analysis

The SPSS 26.0 software was used to analyze the data. Quantitative data with a normal distribution are presented as the mean ± standard deviation, while quantitative data with a non-normal distribution are presented as the median (inter-quartile range). Quantitative data with normal distribution or non-normal distribution were analyzed using the Student’s t-test or the Mann–Whitney U-test, respectively. All count data were analyzed using the Chi-square test or Fisher’s exact test. A two-sided p-value < 0.05 was considered statistically significant.

## Results

**Fig. 2:**
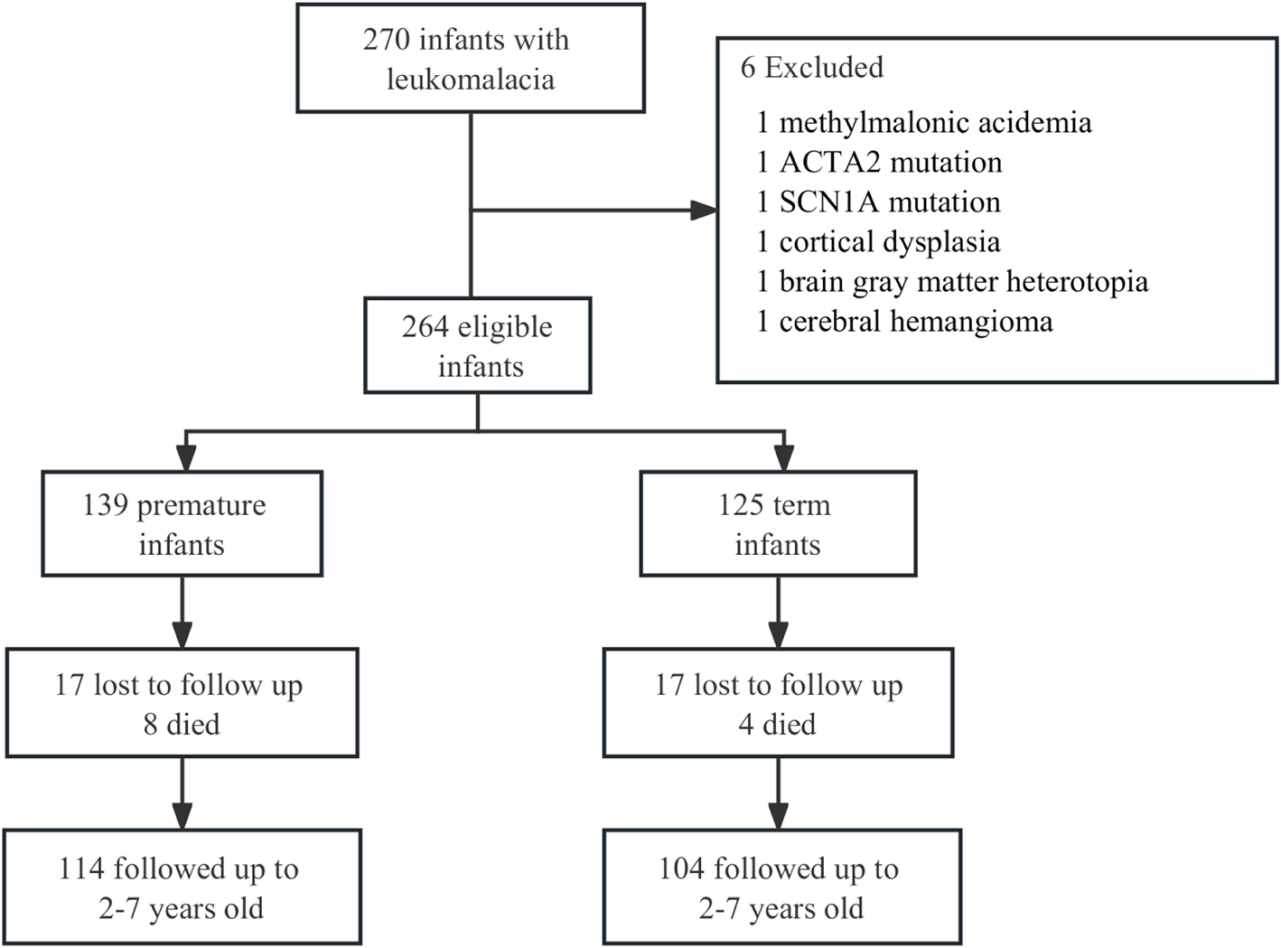
Flow chart of the study participants

During the study period, a total of 270 newborns diagnosed with leukomalacia were enrolled, and 6 infants were excluded, including 3 with inherited metabolic disorders (1 with methylmalonic acidemia, 1 with ACTA2 mutation, and 1 with SCN1A mutation found during follow-up) and 3 with congenital brain malformations (1 with cortical dysplasia, 1 with brain gray matter heterotopia, and 1 with cerebral hemangioma). The remaining 264 eligible infants with leukomalacia were divided into the preterm group (139 infants) and the term group (125 infants). During the follow-up, 17 preterm infants were lost to follow-up and 8 died within the first year (3 died from severe pneumonia, 3 died from sepsis, and 2 died from severe intraventricular hemorrhage), while 17 term infants were lost to follow-up and 4 died within the first year (3 died from sepsis and 1 died from severe pneumonia). At the end of the follow-up, 218 surviving infants, including 114 preterm infants and 104 term infants, were included in the analysis (Fig. 2).

### Comparison of clinical manifestations between the preterm and term groups

The clinical manifestations in the two groups are shown in Table 1. The mean gestational age was 30.6 weeks (range 28.8–33.3 weeks) in preterm infants and 38.7 weeks (range 38.3–39.4 weeks) in term infants. The average birth weight was 1613.7 ± 558.2 g in preterm infants and 3187.6 ± 592.9 g in term infants. The incidence of 5-min Apgar ≤ 7 (χ^2^ = 6.454; *p* = 0.011) and mechanical ventilation (χ^2^ = 23.217; *p* < 0.001) in the preterm group was significantly increased compared to the term group. Preterm infants with leukomalacia were more prone to apnea (χ^2^ = 23.772; *p* < 0.001), sepsis (χ^2^ = 25.757; *p* < 0.001), and hs-PDA (χ^2^ = 4.904; *p* = 0.027), while term infants with leukomalacia were more prone to neonatal seizures (χ^2^ = 20.214; *p* < 0.001) and symptomatic hypoglycemia (χ^2^ = 21.503; *p* < 0.001).

**Table 1:** Clinical manifestations of leukomalacia in the preterm group and the term group

	Preterm group	Term group	*P value*
	(*n* = 114)	(*n* = 104)	
**Basic information**			
GA (weeks)	30.6 (28.8, 33.3)	38.7 (38.3, 39.4)	< 0.001***
BW (g)	1613.7 ± 558.2	3187.6 ± 592.9	< 0.001***
Male	76 (66.7%)	68 (65.4%)	0.824
SGA	18 (15.8%)	12 (11.5%)	0.363
5-min Apgar ≤ 7	42 (36.8%)	22 (21.2%)	0.011*
Cesarean section	72 (63.2%)	56 (53.8%)	0.163
Mechanical ventilation	70 (61.4%)	30 (28.8%)	< 0.001***
**Neonatal complications**			
Apnea	40 (35.1%)	8 (7.7%)	< 0.001***
Neonatal seizures	5 (4.4%)	27 (26.0%)	< 0.001***
Symptomatic Hypoglycemia	5 (4.4%)	28 (26.9%)	< 0.001***
Sepsis	57 (50.0%)	18 (17.3%)	< 0.001***
hs-PDA	10 (8.8%)	2 (1.9%)	0.027*
III-IV IVH	14 (12.3%)	6 (5.8%)	0.096
Cerebral parenchymal Hemorrhage	19 (16.7%)	20 (19.2%)	0.622
Purulent meningitis	12 (10.5%)	7 (6.8%)	0.332

*GA* gestational age, *BW* birth weight, *SGA* small for gestational age, *hs-PDA* hemodynamically significant patent ductus arteriosus, *IVH* intraventricular hemorrhage

^***^p ≤ 0.001; * *p* ≤ 0.05

### Comparison of MRI features between the preterm and term groups

The comparison of the MRI features of leukomalacia in the two groups indicated that the proportion of bilateral distribution of cysts in the preterm group was significantly higher than that in the term group (χ^2^ = 11.547; *p* = 0.001). The major types of leukomalacia were PVL in the preterm group and SCL in the term group, respectively (χ^2^ = 55.166; *p* < 0.001). Grade II PVL accounted for the largest proportion of PVL in both groups (Table 2).

**Table 2:** MRI characteristics of leukomalacia in the preterm group and term group

	Preterm group (*n* = 114)	Term group (*n* = 104)	*P-value*
**Diagnosed age (days)**	30.7 ± 8.0	28.0 ± 9.4	0.024
**Distribution of cysts**			
Unilateral	23 (20.2%)	43 (41.3%)	0.001***
Bilateral	91 (79.8%)	61 (58.6%)	
**Type of leukomalacia**			
SCL	25 (21.9%)	75 (72.1%)	< 0.001***
PVL	89 (78.1%)	29 (27.9%)	
I	11 (9.6%)	6 (5.8%)	
II	58 (50.9%)	15 (14.4%)	
III	20 (17.5%)	8 (7.7%)	

*PVL* periventricular leukomalacia, *SCL* subcortical cystic leukomalacia

^***^p ≤ 0.001

Among the 28 infants with grade III PVL, cysts were located in the parietal lobe in 80% of the preterm infants, while they were located in the temporal lobe in 62.5% of the term infants. In the 100 infants with SCL, cysts were located in the parietal lobe in 64.0% of the preterm infants and in 78.7% of the term infants. However, there was no significant difference between the two groups in the distribution of cysts either in grade III PVL or SCL (Table 3).

**Table 3:** Sites of cysts of Grade III PVL and SCL in the preterm and term groups

	Grade III PVL			SCL		
Sites	Preterm group (*n* = 20)	Term group (*n* = 8)	*P value*	Preterm group (*n* = 25)	Term group (*n* = 75)	*P value*
Frontal lobe	8 (40.0%)	4 (50.0%)	0.691	13 (52.0%)	36 (48.0%)	0.729
Temporal lobe	10 (50.0%)	5 (62.5%)	0.686	15 (60.0%)	35 (46.7%)	0.248
Parietal lobe	16 (80.0%)	4 (50.0%)	0.172	16 (64.0%)	59 (78.7%)	0.142
Occipital lobe	8 (40.0%)	4 (50.0%)	0.691	12 (48.0%)	45 (60.0%)	0.294
Basal ganglia	4 (20.0%)	1 (12.5%)	1.000	4 (16.0%)	9 (12.0%)	0.732
Thalamencephalon	2 (10.0%)	/	/	2 (8.0%)	6 (8.0%)	1.000
Brainstem	/	/	/	1 (4.0%)	2 (2.7%)	1.000
Cerebellum	1 (5.0%)	1 (12.5%)	0.497	/	3 (4.0%)	/

Children with cysts in multiple brain regions were counted repeatedly

### Comparison of neurodevelopmental outcomes between the preterm and term groups

Infants were followed up to an average age of 4.2 ± 1.6 years of age. There was no significant difference in the incidence of NDI in the preterm group compared to the term group (χ^2^ = 0.917; *p* = 0.338). However, the incidence of CP in the preterm group was significantly higher than that in term group (χ^2^ = 4.896; *p* = 0.027), and the incidence of ID (χ^2^ = 9.445; *p* = 0.002) and EP (χ^2^ = 23.049; *p* < 0.001) in the preterm group was significantly lower than that in term group. The most common EP seizure type was West syndrome in both groups (Table 4). Among the infants with CP, the rate of spastic diplegia in the preterm group was significantly higher than that in the term group (χ^2^ = 14.051; *p* < 0.001), while the rate of spastic hemiplegia in the term group was significantly higher than that in preterm group (χ^2^ = 7.376; *p* = 0.007) (Table 5).

**Table 4:** Neurodevelopmental outcomes in the preterm and term groups

	Preterm group (*n* = 114)	Term group (*n* = 104)	*P value*
Follow-up age	4.0 ± 1.5	4.4 ± 1.5	0.071
NDI	92 (80.7%)	89 (85.6%)	0.338
CP	86 (75.4%)	64 (61.5%)	0.027*
GFMCS			
I—III	41 (36.0%)	38 (36.5%)	0.156
IV—V	45 (39.5%)	26 (25.0%)	
ID (GQ < 70)	41 (36.0%)	59 (56.7%)	0.002**
EP	21 (18.4%)	51 (49.0%)	< 0.001***
West syndrome	14 (12.3%)	29 (27.9%)	
Partial seizures	3 (2.6%)	11 (10.6%)	
Generalized seizures	4 (3.5%)	9 (8.7%)	
Mixed seizures	0	2 (1.9%)	
Vision problem	13 (11.4%)	10 (9.6%)	0.668
Strabismus	9 (7.9%)	6 (5.8%)	
Amblyopia	4 (3.5%)	1 (0.9%)	
Blindness	0	3 (2.9%)	
Deafness	1 (0.9%)	/	/

*NDI* neurodevelopmental impairment, *CP* cerebral palsy, *GMFCS* gross motor function classification system, *ID* intellectual disability, *GQ* General Quotient, *EP* epilepsy

^***^p ≤ 0.001; ** *p* ≤ 0.01; * *p* ≤ 0.05

**Table 5:** Different types of CP in the preterm and term groups

	Spastic quadriplegia	Spastic diplegia	Spastic hemiplegia	Other
Preterm group (*n* = 86)	27 (31.4%)	29 (33.7%)	28 (32.6%)	2 (2.3%)
Term group (*n* = 64)	21 (32.8%)	5 (7.8%)	35 (54.7%)	3 (4.7)
*P value*	0.854	< 0.001***	0.007**	0.651

Other: dyskinesia, ataxia, hypotonia, and mixed CP

^***^p ≤ 0.001; ** *p* ≤ 0.01

The Incidence of simple CP in the preterm group was significantly higher than that in the term group (χ^2^ = 10.109; *p* = 0.001). The incidence of CP combined with ID and EP (χ^2^ = 4.122; *p* = 0.042) in the term group was significantly higher than that in preterm group (Table 6).

**Table 6:** Neurodevelopmental impairment comorbidities in the preterm and term groups

	CP	ID	EP	CP + ID	CP + EP	ID + EP	CP + ID + EP
Preterm group (*n* = 114)	43 (37.7%)	3 (2.6%)	1 (0.9%)	19 (16.7%)	3 (2.6%)	/	17 (14.9%)
Term group (*n* = 104)	19 (18.3%)	3 (2.9%)	6 (5.8%)	13 (12.5%)	3 (2.9%)	15 (14.4%)	27 (26.0%)
*P value*	0.001***	1.000	0.056	0.385	1.000	/	0.042*

*CP* cerebral palsy, *ID* intellectual disability, *EP* epilepsy

^***^: *p* ≤ 0.001; * *p* ≤ 0.05

**Fig. 3:**
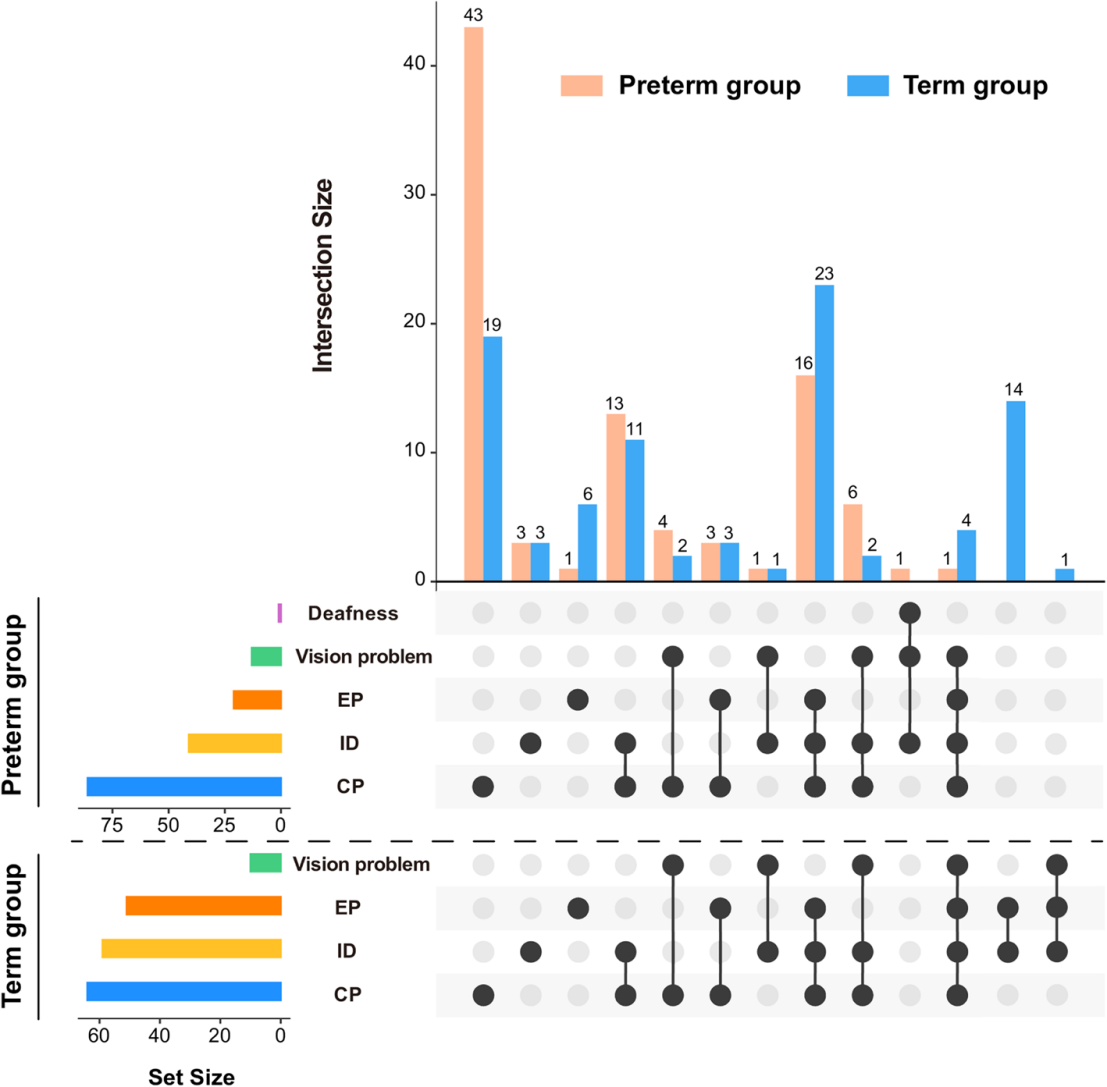
Upset plot of NDI complications in the preterm and term groups. CP: cerebral palsy; ID: intellectual disability; EP: epilepsy

The upset plot of the NDI comorbidities in the two groups was drawn using the UpSetR R package [22] (Fig. 3). The horizontal bar plot in the bottom-left corner shows the sizes of different types of NDI in the two groups. The matrix layout at the bottom shows the NDI comorbidities by showing which sets are intersected. The main bar plot shows the sizes of the NDI comorbidities that were defined by the respective intersections. For example, the first and second bars indicate that there were 43 preterm infants with simple CP and 19 term infants with simple CP. The seventh and eighth bars indicate there were 13 preterm infants and 11 term infants with both CP and ID.

## Discussion

We conducted a prospective cohort study in neonates with leukomalacia with long-term follow-up to 2–7 years of age and found that there were obvious characteristic differences in clinical manifestations, MRI imaging features, and long-term neurodevelopmental outcomes between preterm infants and term infants with leukomalacia. Our study thus provides a more comprehensive understanding of leukomalacia in newborns compared to previous studies.

In terms of clinical manifestations, preterm infants had higher incidence of 5-min Apgar score ≤ 7, mechanical ventilation, apnea, hs-PDA, and sepsis. These complications were mainly related to hypoxia–ischemia and inflammation. Several previous studies have reported that the white matter in preterm infants is more sensitive to the damage caused by hypoxia–ischemia and inflammation than the white matter in term infants [8, 23]. White matter damage in preterm infants is closely related to the susceptibility of premyelinating oligodendrocytes (pre-OLs) to injuries caused by hypoxia–ischemia and inflammation. With the development of the white matter, pre-OLs gradually differentiate into immature OLs (initiation of myelination) and mature OLs (completion of myelination), and their resistance to hypoxia–ischemia gradually increases. In addition, due to the delayed expression of antioxidant enzymes including superoxide dismutase-1 and 2, catalase, and glutathione peroxidase, the white matter in preterm infants may be more susceptible to oxidative damage than in term infants [24].

This study showed that symptomatic hypoglycemia was more likely to occur in the term group, which was similar to a study conducted in Turkey [25]. They found that among 110 children with hypoglycemia, 25 children had brain injuries, including leukomalacia, according to head MRI. Importantly, only 2 of them were preterm newborns and the rest were term newborns or older, suggesting that hypoglycemia is one of the important causes of leukomalacia in term infants. It has been well recognized that hypoglycemia can increase the risk of brain injury [26], but whether hypoglycemia-induced brain injury is more likely to occur in term infants is still not clear. There are a couple of possible reasons for this. First, compared with preterm infants (especially very low birth weight infants) who arrive in the NICUs immediately after birth and receive standardized blood glucose monitoring and timely treatment, term infants are mostly fed at home by breast milk. If the breast milk is not sufficient, term infants can face the risk of hypoglycemia in the first days after birth due to poor feeding [13]. Second, the immature brain can tolerate lower blood glucose concentrations than the mature brain and is less vulnerable to hypoglycemia [27]. Some animal experiments have also provided evidence for this [28], but further research on this topic is still needed.

We found that the MRI features of leukomalacia in preterm infants were different from those in term infants. Although the cysts in the two groups were mainly bilaterally distributed, the proportion of unilateral cysts in the term group was significantly higher than that in the preterm group. PVL is the main type of leukomalacia in preterm infants, while SCL is the main type of leukomalacia in term infants, which indicates that the brain injury patterns of preterm infants and term infants are different. In preterm infants, the distal region of the perforator artery that supplies the ventricular white matter is very sensitive to hypoxia and ischemia due to immature cerebral vascular development. Once hypoxic-ischemic events occur, the injury will easily involve this region and lead to PVL [29], and the cysts will be mostly located in the bilateral hemispheres of the brain [30]. In the mature cerebrovascular development in term infants, the intervascular boundary region gradually moves away from the periventricular white matter to the cortex and reaches the deep white matter area (between the anterior cerebral artery and the middle cerebral artery and between the middle cerebral artery and the posterior cerebral artery). When hypoxia and ischemia occur, this area becomes more vulnerable to the damage and results in SCL [31, 32]. Cysts in SCL tend to be unilateral, which may due to the asymmetric distribution of the middle cerebral artery and its branches [33]. In addition to the degree of maturity in brain development, the severity and duration of hypoxia–ischemia also contributes to the brain injury, which especially involves the metabolically active regions such as the basal ganglia, thalamus, brain stem, and cerebellum [5]. Interestingly, our study showed that 27.9% (29/104) of the term infants suffered from PVL, which means that term infants might suffer brain injury at an earlier point in time in the uterus [12].

Currently there are only a few studies on the long-term neurodevelopmental outcomes of leukomalacia. Resch [30] and Choi [16] both reported that about 86% of children with PVL have poor neurological outcomes, but their sample sizes were small and only preterm infants were included. In our study, we conducted a long-term follow-up to an average age of 4.2 ± 1.6 years old and found that the overall neurological outcomes in infants with leukomalacia were poor and mainly consisted of CP, ID, EP, vision problems, and deafness. Our study indicated that the incidence of CP was higher in preterm infants than that in term infants. In fact, the main type of leukomalacia in preterm infants is PVL, which commonly impairs the corticospinal tracts and leads to motor dysfunction [34–36]. In addition, several previous studies have shown that PVL complicated with CP in preterm infants tends to result in spastic diplegia [12, 37, 38], and the same result was found in our study. Interestingly, we found that the main type of CP in term infants with leukomalacia was spastic hemiplegia. A systematic review [33] reported that 58 out of 61 (95%) children with CP who were born at full-term were hemiplegic, and this was attributed to the unilateral brain injury in most of these children. This is consistent with the higher proportion of unilateral cysts in term infants compared to preterm infants seen in our study. Moreover, we found that term infants with leukomalacia were more likely to suffer from ID and EP. A possible explanation for this is that the dominant type of leukomalacia in term infants was SCL, which might involve the deep white matter and cortex in several cerebral lobes and impair not only motor development, but also intellectual development [39]. The impairment of subcortical-cortical circuits, specifically affecting the white matter junction between the basal ganglia/thalamus and the cortex, is responsible for disrupted network integration and contributes to motor, attention, language, vision, and memory function disorders [40, 41]. Additionally, cysts play a crucial role in disrupting nerve electrophysiological stability, leading to abnormal excitability of neurons and the development of EP [42, 43]. Furthermore, recurrent seizures can cause damage to brain tissue and further worsen cognitive development [44]. The above reasons also explained the higher incidence of CP combined with ID and EP in the term infants with leukomalacia. We also found that the most common type of EP in both groups was West syndrome, which was consistent with previous studies [45, 46]. Leukomalacia, especially severe PVL [45, 47] and parieto-occipital SCL [46], are known to be the important causes of West syndrome. Additionally, although grade III–IV IVH often leads to poor neurological outcomes [48, 49], there was no difference between the two groups in the incidence of grade III–IV IVH in the surviving children. Thus, the long-term outcome of leukomalacia was not affected by grade III–IV IVH in our study.

There were several limitations to this study. First, there was no significant difference in the regions of the cysts of grade III PVL and SCL between the preterm and term groups, which was probably due to the small sample size. Second, in children who developed West syndrome, whole exome sequencing should be used for further understanding of the disease. Finally, the follow-up to 2–7 years of age was not long enough because both intelligence and language development require longer follow-up to school years. However, at the very least, we have provided clinical evidence for the long-term neurological outcomes of preterm and term infants with leukomalacia.

## Conclusion

We found differences in the clinical manifestations of leukomalacia in term infants and preterm infants, and PVL mainly occurred in preterm infants, while SCL was commonly seen in term infants. Although the long-term neurodevelopmental outcomes of both types of leukomalacia were both poor, preterm infants were more prone to CP, while term infants were more prone to ID, EP, and the combination of CP with ID and EP. Further well-designed prospective multicenter studies with larger sample sizes and with follow-up to school age or longer are still needed to confirm the long-term neurological outcomes of leukomalacia.

## Data Availability

The datasets used and/or analyzed during the current study are available from the corresponding author on reasonable request.

## Abbreviations

MRI: Magnetic resonance imaging
PVL: Periventricular leukomalacia
SCL: Subcortical cystic leukomalacia
NDI: Neurodevelopmental impairment
CP: Cerebral palsy
ID: Intellectual disability
EP: Epilepsy
NICUs: Neonatal intensive care units
hs-PDA: Hemodynamically significant patent ductus arteriosus
IVH: Intraventricular hemorrhage
GMFCS: Gross motor function classification system
pre-OLs: Premyelinating oligodendrocytes
